# An Enquiry into the Status of American Foulbrood Therapeutics

**DOI:** 10.3390/insects17030312

**Published:** 2026-03-13

**Authors:** Olivia Ducommun-Dit-Verron, Gemma Zerna, Travis Beddoe

**Affiliations:** AgriBio, Centre for AgriBioscience, Department of Ecology, Plant and Animal Science, School of Agriculture, Biomedicine and Environment, La Trobe University, 5 Ring Rd, Bundoora, VIC 3083, Australia; o.ducommun-dit-verron@latrobe.edu.au (O.D.-D.-V.); g.zerna@latrobe.edu.au (G.Z.)

**Keywords:** American foulbrood, therapeutics, *Paenibacillus larvae*, vaccine

## Abstract

Managed colonies of the Western honey bee, *Apis mellifera*, are essential to global food security by ensuring the pollination of a wide array of crops that are crucial for human consumption. However, substantial declines in managed honey bee populations have been reported worldwide, including in Australia, the United States and Europe. These losses have been attributed to the multifaceted interplay of stressors encompassing agrochemical impact, climate fluctuations, pathogens, suboptimal forage conditions, and habitat reduction. In particular, *Paenibacillus larvae*, the causative agent of American foulbrood (AFB), is one of the most destructive bacterial pathogens for honey bees due to its high transmissibility, environmental persistence, and capacity to cause complete colony collapse. Recurrent and widespread AFB outbreaks impose significant economic and biosecurity burdens on apiarists, exacerbating declines in pollination services and agricultural productivity. This review synthesises the current landscape of therapeutic strategies targeting AFB, including bacteriophage-based approaches, vaccine development, probiotics, and essential oils, and evaluate their reported field applications, efficacy, and practical limitations. Bacteriophages and immune-priming approaches show the greatest potential to reduce larval mortality and pathogen load, although their application is constrained by formulation stability, delivery challenges, and limited large-scale field validation. Probiotics and essential oils produce highly variable and inconsistent effectiveness under field conditions. Overall, these alternatives currently represent promising complementary tools rather than standalone treatments, underscoring the need for further investigation.

## Simple Summary

Honey bees are vital for pollinating crops that sustain global food production. Yet their populations are declining worldwide due to multiple stressors such as pesticide exposure, climate change, habitat loss, and increasing disease outbreaks. One of the most destructive diseases globally is American foulbrood, caused by a bacterial pathogen that devastates bee colonies and threatens agricultural productivity. This review explores alternative strategies to antibiotics for managing this disease, including bacteriophages, vaccines, probiotics, and plant-derived compounds like essential oils. We summarise how these approaches work, their practical applications, and the challenges that remain. By identifying effective and sustainable solutions, this research aims to support healthier bee populations, reduce reliance on antibiotics, increase the safety of bee products and safeguard pollination services essential for global food security.

## 1. Introduction

The honey bee industry emerges as a cornerstone within the global economy, boasting a valuation in the billions of dollars and overseeing a vast network of managed hives distributed worldwide. In Australia alone, the apicultural industry contributes significantly, with a valuation exceeding $14 billion annually [1]. Integral to the agricultural landscape, honey bees are industrious pollinators that fulfil a crucial ecological role, facilitating the pollination of a multitude of plant species essential for food production and ecosystem stability. A key example is almonds, which are almost entirely dependent on honey bees for pollination [2,3]. Yet, amidst their vital contributions, managed populations of the Western honey bee, *Apis mellifera*, face a host of challenges, with the foremost among them being the continuous presence of pathogens. Within this complex ecosystem, certain pathogens proliferate with alarming frequency, posing significant threats to bee health and agricultural resilience [4].

The Gram-positive spore-forming bacterium *Paenibacillus larvae* is the most significant bacterial pathogen for honey bees [5]. The *P. larvae* pathogen is the causative agent of American foulbrood (AFB), exhibiting high pathogenicity and global distribution [5,6,7,8]. Its eradication is notably challenging, primarily due to its long-lived spores [5]. AFB has been described as one of the most detrimental yet unresolved problems in beekeeping globally [4]. AFB attains the status of a notifiable disease in many countries due to its pronounced infectivity, lethal nature, and the longevity and survivability of its spores [4]. As such, treatment measures are generally regulated by local biosecurity acts or veterinary policies, which consider the incineration of clinically evident diseased colonies to be the only truly effective control measure against infections [5,8]. Upon the detection of AFB in Australia, there exists a legal obligation to report AFB cases, followed by mandatory hive incineration as stipulated by official regulations [1]. The burning of the hive and all the associated equipment results in a huge economic loss [4]. Ultimately, *P. larvae* poses a great threat worldwide to honey bees, beekeepers and subsequently the production of direct and indirect agricultural products [3].

Historically, AFB outbreak management relied solely upon the use of antibiotics such as oxytetracycline and sulfathiazole [5]. However, several problems arise when using antibiotics to prevent and treat AFB [5]. Antibiotics address the clinical symptoms of AFB by inhibiting the replication of the bacterial vegetative forms. Despite this, they prove ineffective in eradicating *P. larvae* spores, rendering them inadequate for the complete inhibition of the AFB infection cycle [9,10]. Consequently, the ineffective use of antibiotics as a treatment for AFB exhibits disastrous effects on beekeeping, bee colonies and agriculture due to the increasing prevalence of antibiotic resistance and residue accumulation within honey bee products [2,3]. The utilisation of antibiotics for AFB treatment and prevention is restricted in numerous countries, including Australia and most European countries, due to legislation that prohibits the presence of antibiotic residues in bee products [2,11,12]. This prohibition is in response to the proliferation of antibiotic-resistant strains, recently described in many parts of the world [10,13,14,15]. The detection of antibiotic residues results in a reduction in honey quality and safety, due to the serious threat of residue accumulation in bee products commonly consumed by humans [2,13]. It has also been observed that an increased frequency of antibiotic-resistant strains of *P. larvae* has arisen due to the extensive overuse of antibiotics, resulting in their overall ineffectiveness [13]. Despite the historic use of antibiotics, hive incineration remains the sole effective measure in controlling AFB.

With the escalating prevalence of incurable bacterial infections and their substantial economic burden precipitated by the mounting challenge of antibiotic resistance, there exists a pressing global need for alternative therapeutic strategies for AFB. This review will outline the current developments in AFB research with a prominent focus on application as well as the limitations of these treatments for the prevention and treatment of AFB.

## 2. Honey Bee Immunity

Honey bees, like many insects, have a robust immune system comprising diverse mechanisms to defend themselves against microbial and eukaryotic pathogens [16,17]. While lacking an adaptive immune system, insects exhibit numerous parallels to the innate immune responses observed in humans and other vertebrates. These similarities include physical barriers, as well as cellular and humoral responses [18].

It is important to distinguish between immune responses directed against viral and bacterial pathogens in honey bees, as these differ mechanistically and are relevant to distinct disease contexts [16]. Antiviral immunity is predominantly intracellular and mediated by RNA interference (RNAi), whereby viral double-stranded RNA is processed into small interfering RNAs that direct sequence-specific degradation of viral genomes [19]. This mechanism is particularly relevant in adult bees, where viral infections commonly replicate within host cells [19]. In contrast, bacterial infections such as AFB involve extracellular, gut-associated stages in larvae and therefore rely primarily on barrier defences, antimicrobial peptides, and hemocyte-mediated responses rather than RNAi-mediated pathways [17,19].

The physical barrier acts as the bee’s first line of defence and consists of the exoskeleton cuticle and the peritrophic membrane lining the digestive tract, which each work to prevent the initial entry of pathogenic organisms into the body cavity [16]. During AFB infection, spores are ingested by larvae and germinate within the midgut [19]. While the peritrophic matrix limits microbial contact with epithelial cells, it does not eliminate spores, which remain highly resistant to environmental and physiological stressors. As a secondary defence mechanism, the innate immunity of honey bees encompasses two main categories: cellular and humoral immunity [16,19]. Recognition of pathogen-associated molecular patterns (PAMPs) by pattern recognition receptors activates conserved signalling pathways, including Toll and immune deficiency (Imd), which regulate the production of antimicrobial peptides (AMPs) such as abaecin, apidaecin, hymenoptaecin, and defensin [2]. These AMPs circulate in the hemolymph and target vegetative bacterial cells. Cellular immunity, mediated by hemocytes, contributes further through phagocytosis, nodule formation, encapsulation, and melanisation via the phenoloxidase cascade [16,17,19]. These mechanisms are effective against metabolically active bacteria but largely ineffective against dormant *P. larvae* spores, which can persist and germinate prior to robust immune activation [17,18,20]. Vitellogenin (Vg) is additionally an essential part of humoral immunity in honey bees [19,21,22]. Synthesised in the fat body within the abdomen of female honey bees, this egg yolk protein is released into the hemolymph and transported to the ovaries and other tissues delivering nutrients as well as immune elicitors to developing eggs [22,23]. Vg is commonly associated with egg production in queen bees and has functions that also protect bees against oxidative stress, which in turn has been indicated to increase the life span of their progeny [22,24]. Studies suggest that Vg acts as an immunomodulatory agent, influencing the bees’ immune response by regulating the production of antimicrobial peptides and enhancing the activity of immune cells such as hemocytes [21,24]. Vg’s multifunctional role additionally extends its utility in vaccine development and antimicrobial strategies. Researchers have explored the potential of Vg in enhancing the immune response in honey bees, providing insights into novel vaccine formulations including *P. larvae*. In turn, harnessing Vg’s immunomodulatory and antimicrobial properties holds promise for developing innovative approaches to protect honey bee populations from disease and enhance their resilience against environmental stressors.

Social immunity is additionally present in honey bees, which involves collective behaviours to prevent the spread of diseases within the colony [17,19,25]. This collective defence against pathogens arises from coordinated actions by multiple individual bees that engage in tasks that collectively reduce the transmission of pathogens and parasites from the hive. For instance, worker bees actively remove diseased brood and deceased adults from the hive [26]. Additionally, adult bees that intentionally die outside of the hive contribute to social immunity by limiting pathogen exposure, particularly when their deaths result from high pathogen loads [23]. Despite this, social immunity limits transmission, but it does not eliminate environmentally persistent spores, which can remain infectious for decades [5].

Although honey bees possess a complex immune system, many of its functions remain underexplored. Despite this complexity, the absence of an adaptive immune system renders honey bees particularly vulnerable to persistent and lethal infections, further emphasising the need for external therapeutic interventions. Understanding the intricacies of these immune pathways is essential for developing novel treatments and vaccines that leverage these natural defence mechanisms. Furthermore, understanding the collective behaviours of social immunity presents an additional opportunity for emerging therapeutics. By recognising the limitations of the honey bee’s immune system and further understanding and utilising the mechanisms in which they collectively defend against pathogens within the colony, researchers can potentially create innovative approaches to enhance bee health and resilience against environmental challenges.

## 3. Current Treatments

### 3.1. Vaccines

Recent studies involving many species of insects have expanded the knowledge of immunology in invertebrates, particularly in honey bees. It was previously believed that honey bees relied purely on their innate defences to combat infection. Research, however, has revealed that the immune system of honey bees shares many similarities to that of vertebrates functioning with cellular and humoral responses such as haemocytes [16,17]. Although the immune system of honey bees lacks antibodies, it does produce immune effectors, for example AMPs, which generate a functional immune response following pathogen exposure [19]. Ultimately, studies in many insect species reveal that encounters with pathogens can enhance the phagocytic activity of haemocytes, leading to the meditation of specific immune protection [16,19]. Furthermore, maternal immunity has been observed, wherein an immune response can be passed down to offspring, positively influencing their pathogen resistance and survival, known as trans-generational immune priming (TGIP) [23,27]. Advances in the understanding of the immune pathways of honey bees are important for the development of novel therapeutics for the prevention and treatment of many pathogens.

The first demonstration of the TGIP phenomenon in honey bees was conducted by López et al. [27], where honey bee queens were injected with heat-killed *P. larvae* bacteria, and the subsequent offspring of these pathogen-injected queens were challenged with *P. larvae* spores (Table 1). The mortality rates of the offspring were measured and found that offspring from primed queens were 26% more likely to survive when faced with infection. However, as discussed by López et al. [27], this method of injecting the queen has many practical limitations and may be harmful, as injury and stress have been found to have considerable effects on the queen bee’s survival [27]. Additionally, at the time of this study, the known mechanism of TGIP in honey bees was yet to be elucidated. However, this mechanism was later uncovered by Salmela et al. [23], who, through immunofluorescence microscopy and Western blotting, identified an egg yolk protein, Vg, as the carrier of immune-priming signals. This work provided an explanation for TGIP in honey bees, further elucidating the previously undescribed role of the Vg protein. This finding opened the possibility of vaccination in honey bees to protect them against many diseases. Dickel et al. [28] conducted the first efficacy vaccine trial, employing a heterologous challenge to demonstrate the safety and protective effects of orally administering an inactivated *P. larvae* bacterin to queen bees. This intervention induced protection in subsequent generations of larvae [28]. As summarised in Table 1, oral delivery of the inactivated pathogen to queens resulted in a significant decrease in AFB infection in progeny by 30–50% under laboratory conditions following vaccination. It is important to note that hives lacking clinical symptoms can harbour AFB spores. Dickel et al. [28] observed a tolerance level with up to approximately 158 spores per bee within an unvaccinated hive that had no notable clinical signs of disease. However, Erban et al. [29] found that a spore increase of approximately 30% can trigger a clinical outbreak within a hive. Thus, a 30% or greater decrease in spore level may effectively prevent disease manifestation. Furthermore, continual suppression of hive pathogen load to a tolerable subclinical level could prevent outbreaks from occurring. Consequently, the use of vaccines, even with moderate efficacy, holds promise as a management strategy for controlling infections.

The vaccination approach has become increasingly popular, showing strong potential in protecting colonies from AFB outbreaks; however, the effects of priming on the entirety of the hive must be considered. Table 1 summarises the current applications of TGIP in bees. It is widely accepted that high immune function is often a trade-off against other traits [30]. Consequently, priming the colony may result in undesirable side effects. This was, however, disputed in a two-year study conducted by Leponiemi et al. [31] in 2023 where various hive health parameters were investigated post-priming of the queen bee using inactivated *P. larvae* under natural hive conditions outside the laboratory. Such parameters used to evaluate the hive included hive weight, amount of brood, adult bees, and honey yield, all of which remained unaffected. Additionally, gene expression in the larvae was also investigated; this again remained unchanged compared to non-primed colonies. Subsequently, the authors concluded that no significant trade-offs occurred due to TGIP [31]. These results have important practical applications in exploiting TGIP as a safe tool in fighting against AFB.

**Table 1:** Current application of inactivated *P. larvae* bacterin vaccines against *P. larvae* in honey bees.

Treatment	Results	Reference
Injection of heat-killed *P. larvae* bacterin at a concentration of 10^8^ bacterial cells per ml to the queen bees followed by the offspring challenged with *P. larvae* spores. Number of dead larvae was recorded each day for the duration of the trial (*n* = 12 days). Trial was repeated in 2011 and 2012.	Cumulative larval mortality found a significant 26% reduction in larval mortality in primed queens in both years the trial was performed.	[27]
Oral administration of inactivated *P. larvae* vegetative bacterin to 47 queen bees over an 8-day period and placed in hive across two locations. Offspring of the primed queens were reared in a laboratory setting and challenged twice with *P. larvae* to a final concentration of 10,000 spores/379 µL. Number of dead larvae were removed and recorded over an 8-day period.	Mortality was significantly decreased by 30–50% in *P. larvae*-challenged larvae from *P. larvae* bacterin-vaccinated colonies compared to placebo hives across the two locations.	[NO_PRINTED_FORM]
Oral administration of bacterin to the queen bees over a 6-day period followed by assessment of pathogen presence, hive parameters and gene expression over two seasons in a natural environment.	No effect from TGIP on any hive metrics including hive weight parameters, pathogen presence and gene expression in offspring.	[31]
Queenless colonies containing only brood and nurse bees were fed a supplement containing inactivated *P. larvae* bacterin over a 3-day period. Newly deposited royal jelly was harvested for analysis via fluorescence microscopy or proteomic analysis.	Bacterial fragments found to be incorporated in royal jelly samples from pathogen-diet colonies. Larvae were found to have significantly higher levels of the antimicrobial peptide found in royal jelly, defensin-01.	[25]

Recent research has additionally shed light on how queen bees transfer pathogen fragments into their eggs via Vg [22,23]. This egg yolk precursor protein is also used by nurse bees to synthesise royal jelly [25]. Royal jelly has therefore been proposed as a potential vehicle for bacterial fragment transfer between nurse bees, queens, and larvae [25,32]. In an exploratory study, pathogen fragments ingested by nurse bees were transported to their jelly-producing glands. These bacterial fragments were subsequently found to be incorporated into the royal jelly [25]. Moreover, when larvae consumed the royal jelly-containing pathogen cells, it resulted in higher production levels of the antimicrobial peptide, defensin-1 [25]. These findings underscore the multifaceted roles of royal jelly in honey bee immunity, as well as colony health, whilst providing an additional avenue for vaccine development.

#### Limitations of Vaccines

While TGIP has been demonstrated in honey bees, these studies rely on inactivated bacteria to be injected or orally administered to a bee, which presents an array of limitations. Inactivated bacterial cell vaccines have been widely studied for prophylactic treatment against infectious disease [32]. It is important to note that these vaccines may contain bacterial toxins, which can serve as a crucial component in their efficacy; however, they also pose potential liabilities in terms of safety, regulatory compliance such as the Good Manufacturing Practices (GMPs), and public perceptions. Alterations in the inactivation procedure may address these liabilities; however, these may directly affect the function and ability of the vaccine to perform effectively. Thus, selection of the inactivation method as well as its preparation must be acknowledged [32]. Furthermore, the utilisation of inactivated pathogens, despite their inherent advantages, is accompanied by several other limitations. These include a lack of immune response, the potential need for multiple doses, and variations in antigen presentation and pathogen diversity [32,33]. Although the research conducted by Dickel et al. [28] has now resulted in a USDA-conditionally-approved vaccine commercially available through Dalan Animal Health (Athens, GA, USA), there remains a critical gap in understanding the efficacy of this vaccine outside laboratory conditions.

Key considerations regarding the use of inactivated vaccines in real-world hive conditions include environmental variability, bee behaviour and colony dynamics, pathogen exposure, hive health and stress, as well as long-term effects [18,19]. While this initial progress is promising, further research with practical implementation, such as large-scale trials, is required to validate the vaccine’s effectiveness beyond the controlled confines of the laboratory. Furthermore, the Dalan Animal Health (Athens, GA, USA) vaccine demonstrated an efficacy range of 30–50% as detailed in Table 1 [28]. While a 30% or greater reduction in spore levels has been shown to prevent disease manifestation, additional research revealed a critical association between spore counts and colony survival [20]. Using forty honey bee colonies, Stephan et al. [20] inoculated the same dose of *P. larvae* spores to induce AFB epidemics. The cascading impact of the spores on brood mortality led to a gradual decline in adult worker bee numbers in all colonies, ultimately leading the colony into an unrecoverable negative population spiral [20]. Despite this, the data produced by Dickel et al. [28] indicates that the innate immune response in honey bees is sufficient to reduce the spore count to levels that significantly impact clinical disease manifestation. However, large-scale longitudinal field efficacy trials are necessary to understand the overall effects of oral vaccination on colony health. While the advancement of vaccine development offers significant promise for managing bee health and mitigating outbreaks of infectious diseases, it is essential to explore complementary approaches of therapeutics, such as phage therapy and other antimicrobials.

### 3.2. Bacteriophages

Bacteriophages (phages) are bacterial viruses that play a dominant role in controlling bacterial populations and their therapeutic use has the potential to overcome the disadvantages associated with antibiotics [34,35]. A single type of bacteria will have multiple phages that specifically bind and infect only that bacterium [36,37]. Phages can be categorised into two primary types: lytic phages [36,37] and temperate phages [35,36]. Lytic phages replicate through the lytic cycle and naturally cause bacterial lysis as a part of their regular lifecycle [35]. Conversely, temperate phages undergo the lysogenic lifecycle, during which the phage’s DNA is incorporated into the bacterial genome resulting in the production of a prophage [35,38]. This viral genetic material, known as a prophage, can persist in this state for generations, until an environmental signal triggers mechanisms that excise the prophage material from the bacterial genome, initiating the lytic cycle [35]. Temperate phages are additionally recognised for their active role in facilitating horizontal gene transfer, a key mechanism involved in the spread of antimicrobial resistance and virulence genes [39]. As a result, temperate phages are not considered as suitable candidates for phage therapy.

AFB management has historically relied on antibiotics such as oxytetracycline and sulfathiazole; however, these treatments only suppress vegetative *Paenibacillus larvae* and fail to eradicate highly resilient spores, leaving the infection cycle intact [34,40]. Continued antibiotic use has contributed to the global emergence of antibiotic-resistant *P. larvae* strains and residue accumulation in honey bee products, with negative impacts on beekeeping and agriculture [5,15,41]. Consequently, phage therapy is emerging as a promising alternative, offering a targeted approach to controlling AFB while addressing antibiotic resistance and residue concerns [38,42,43]. The isolation of lytic phages for therapeutic use is relatively simple, inexpensive, and a rapid process. Therefore, due to the widespread emergence of multi-drug-resistant bacterial pathogens, bacteriophages are seen as promising antimicrobial agents against spore-forming bacteria [34,44]. *P. larvae*, like all bacterial species, possess these natural opponents. A range of studies have examined these bacteriophages and concluded that they are commonly found in the hive environment [38,43,45,46]. The first *P. larvae* bacteriophage was isolated from dead larvae in the 1950s at The Leningrad Veterinary Institute, Russia [38], although it was not until 2013 when the first *P. larvae* phage (philBB_Pl23) whole genome was sequenced [47]. Since this time, a further 48 *P. larvae* phage genome sequences have been obtained [38,43]. Sources for *P. larvae* phage isolation include infected larvae, bee products, hive elements, soil and water from around the hive, as well as lysogenic bacteria [38,43,45,48]. Furthermore, as a food additive, the US Food and Drug Administration (FDA) regards phages as safe for human consumption [49]. Thus, the field of bacteriophage therapy presents a promising avenue for addressing the persistent threat of AFB in apiculture worldwide.

Since the first isolation of phages that are active against *P. larvae*, many further bacteriophages have been isolated and described; however, only a select few have been analysed for their therapeutic activity [46,50,51,52]. Table 2 presents selected data outlining the current *P. larvae* phage application in bees. Researchers have conducted in vitro studies to assess the impact of *P. larvae* phages on infected larvae [46,50,52]. In two of these studies, larvae infected with *P. larvae* were subsequently treated with phages and exhibited a significantly higher survival rate compared to untreated infected larvae [46,52]. Importantly, uninfected larvae exposed to phages had very similar survival rates to the control larvae, indicating the phages had no adverse effect on the honey bee larvae or their gut microbiota [46,52]. Similar promising outcomes were observed by Ghorbani-Nezami et al. [46] in hives where larvae treated with phages displayed higher survival rates post-*P. larvae* infection with no adverse effect on the larvae. Additionally, the phages (termed F, WA and XIII) used also had a prophylactic effect, where larvae that were treated with phages and subsequently infected had significantly higher survival compared to larvae treated post-infection [46]. Furthermore, the phage API480, characterised from hive soil in Spain by Ribeiro et al. [53], exhibited a broad lytic spectrum against *P. larvae* strains and was active against 69% of the strains tested in vitro. Despite the API480 phage being characterised as temperate, its strong lytic activity suggests it could be a viable candidate for application in hives to treat or protect honey bees in field conditions. Additionally, lysis assays were performed on closely related bacterial species and revealed the inability of API480 to infect these bacteria [53]. Brady et al. [51] demonstrated that a phage cocktail active against *P. larvae* may be effective when used both prophylactically and therapeutically (Table 2). This study selected three phages to create a cocktail according to the combined phage’s ability to lyse all tested strains of *P. larvae*, as outlined in Table 2. The efficacy of phage treatment was evident from the outcome that all 24-phage treated infected hives recovered from AFB infection [51]. Safety verification additionally confirmed that the phage cocktail did not adversely affect the mortality of treated bees, even after an overdose was administered [48]. In contrast, the phage HB20c2 investigated by Beims et al. [50] did not improve the survival rate of infected larvae, despite a lytic ability *in vitro*, directly against *P. larvae*. This observed discrepancy may arise due to the phage either entering lysogeny or facing challenges in entering the honey bee gut, where the *P. larvae* infection occurs [40,50]. Moreover, numerous studies have found phages to be highly specific to *P. larvae* as they failed to lyse closely related bacterial species such as *P. lentimorbus*, *P. polymya* and *P. aveli* [46,50,52]. This high specificity of bacteriophages as antimicrobials is a major advantage for therapeutic use [36]. In turn, they ensure that the regularly found microbiota remains undisturbed during phage therapeutic use [36,37].

By contrast, antibiotics can have major effects on microbiota namely due to their broad mode of action [37,38]. The Bonilla-Rosso et al. [54] study investigated the association between the gut microbiota of honey bees and phages and found that phages are a natural part of the honey bee’s microbiome. However, the authors suggested that further studies should be conducted to advance our understanding of phage–bacteria interactions as well as their specific role in bee gut microbiota and their overall health [54]. Additionally, a study conducted by Brady et al. [48] suggested that *P. larvae* phages may bind to the spore and vegetative forms of the bacteria, providing a unique feature in treatment against AFB. Despite the current published data suggesting phage application does not cause any adverse effects on the honey bee, larvae, or products for human consumption, further research is urgently required for an effective and safe product to treat AFB.

#### 3.2.1. Endolysins

Recently, there has been growing interest in utilising phages to control *P. larvae* infections. However, the isolation of temperate phages rather than lytic phages has hindered the progression of phage therapy [7]. Nevertheless, the identification of the lytic machinery within phage sequences is regarded as a promising approach to overcome this barrier. Of interest are phage-encoded enzymatic proteins, termed ‘endolysins’ or ‘lysins,’ which are synthesised by bacteriophages during the final stages of their replication cycle and act by hydrolysing the host cell wall, leading to the degradation of the bacterial peptidoglycan layer and subsequent bacterial lysis [55]. Endolysin treatment has several advantages over conventional antibiotic therapy, including rapid host killing, high host specificity, preservation of normal microflora, efficacy against multi-resistant bacteria and synergy with other antibacterial agents [38,55]. These key biological aspects are particularly relevant for *P. larvae* control, where spore presence and limited therapeutic options present major challenges. Notably, their specificity and ability to target drug-susceptible and drug-resistant organisms make them attractive as antibacterial agents [55]. Endolysins often display broader antibacterial activity than intact bacteriophages because they directly target conserved peptidoglycan structures and do not require host-specific receptor recognition [55,56]. Numerous studies have previously characterised many endolysins through recombinant methods, demonstrating their efficacy in successful inhibition of bacterial growth in vitro and in vivo [56,57,58]. Table 3 presents the current data outlining the use of *P. larvae* phage endolysins to combat AFB. The first *P. larvae* bacteriophage endolysin was explored by Oliveira et al. [59]. By employing in silico analysis, the study identified a predicted endolysin from its previously characterised *P. larvae* bacteriophage, phiIBB_Pl23, and further assessed its potential to control AFB infections [59]. In their initial studies, phage philBB_Pl23 was active and able to lyse 16 of the 20 *P. larvae* strains; however, in comparison, its endolysin, PlyPl23, was active against all tested *P. larvae* strains [47,59]. PlyPl23 was also assessed against closely related species of *Bacillus* and *Lactobacillus* and were seen as not active [59]. This further supports endolysins as a potential broad-application antimicrobial highly specific to AFB. Additionally, the in vivo safety evaluation assay demonstrated that this endolysin was not toxic to the bee larvae and therefore did not affect microbial activity within the larvae gut. Similarly, LeBlanc et al. [60] isolated a novel *P. larvae* bacteriophage lysin, PlyPalA, and found the lysin to be highly lytic against pathogenic *P. larvae* strains. Moreover, the authors evaluated the endolysin lytic activity using a wide range of biochemical conditions and found that it protected larvae in vivo from lethal infection and did not harm commensal bacteria known to be present in honey bee larval microbiota [60]. These features indicate that phage lysin seems to be a better candidate than whole phages for preventing and eliminating *P. larvae* infections [46,52,60]. Endolysins, therefore, present a compelling therapeutic option for AFB infections; as a result, the use of a targeted endolysin treatment against *P. larvae* could be beneficial to honey bees and agriculture.

#### 3.2.2. Limitations of Bacteriophages and Endolysins

Despite the considerable potential of phages to be a valuable solution in mitigating AFB with a wide range of positive literature establishing them as a promising solution, their application presents with limitations [46,50,51,52]. Phage efficacy is dependent on various properties such as physiochemical conditions, host physiological conditions, as well as the phage’s ability to penetrate and replicate at the site of infection to become effective [36,38]. Due to the ability of the phage to replicate in the host, it is crucial that selected phages do not exhibit generalised transduction or possess gene sequences with significant homology to antibiotic-resistant genes or other bacterial virulence factors [37]. Therefore, rigorous research is required to determine the therapeutic efficacy and confirm the safety of phage use. While lytic ability is essential for effective phage therapy, the isolation of temperate phages possessing lytic activity are, however, compromised by their prophage status. Prophages can excise themselves from the bacterial genome, which may inadvertently cause harm [38,39]. In turn the isolation of purely lytic phages without a prophage presence presents a significant challenge for use in phage therapy. Additionally, recent data has generated conflicting findings regarding the nature of characterised *P. larvae* phages. Avenues of research suggest that all characterised *P. larvae* phages, including those induced from prophages, exhibit lytic behaviour in vitro [38,44]. Conversely, another study posits that these phages are temperate in nature [45]. These conflicting classifications have caused incompatibilities for some phages to be used as a therapeutic. For example, phages initially identified as lytic phages proved to be induced and not directly isolated from the pathogen as previously thought. The consensus, however, is that all known *P. larvae* phages are temperate and able to lyse their host in vitro [7,38]. Temperate phages are excluded from possible application in AFB treatment as they pose a significant risk in phage therapy, primarily through encoding integrases or transposases [34]. These enzymes facilitate horizontal gene transfer, which can lead to the spread of antibiotic-resistant genes and other virulence factors among bacterial populations. The presence of integrases or transposases in therapeutic phages can result in unintended genetic modifications, potentially giving rise to more virulent or resistant bacterial strains [34]. Moreover, these elements can be detrimental to bee larvae, further highlighting the necessity of excluding temperate phages from phage therapy [34,50]. Additionally, the mechanisms governing the induction of *P. larvae* prophages into the lytic cycle remain incomplete and warrant further exploration. It is also imperative that additional research investigates whether prophages can be activated following the administration of a therapeutic endolysin [61]. As endolysins disrupt the bacterial cell wall, resulting in bacterial lysis, this may lead to the release of prophage DNA from the bacterial host’s genome, potentially triggering the activation and initiation of the lytic cycle [56,61]. This process may result in unintended consequences; therefore, the ramifications of prophage excision within the context of therapeutic outcomes require exploration. As such, it is unclear whether such excision will enhance therapeutic efficacy by further lysing pathogenic strains or potentially hinder outcomes, particularly given the potential for prophages to carry antibiotic-resistant genes that can be disseminated to bacteria when infected by a prophage [62,63]. Therefore, comprehensive research in this area is essential to advance phage therapy.

Additional factors that may interfere with phage activity when phages are intended as therapeutics include host conditions and preparation composition [64,65,66]. Phage efficacy is dependent on its properties, structure, and biology, as well as its dose and manner of application [65,67]. In turn, these features must be considered when phage preparation is evaluated as a therapeutic. Although studies have investigated the limitations of phages, their use in the field is yet to be elucidated. Azeredo [65] found that bacteria may already be resistant to phage therapy and suggested a preparation of various phages to create a cocktail to counteract this factor. The study proposed a phage cocktail containing a spectrum of lytic activity able to be effective against a wide host range. The authors suggested this application to limit the probability of resistance to the phages occurring [65]. Although phages are regarded as inherently safe, there are limitations to their use that require further research, specifically regarding the isolation of purely lytic phages. Cost and scalability represent significant limitations to the widespread adoption of bacteriophage- and endolysin-based therapies, as large-scale production, purification, and quality control processes are resource-intensive [35,37,65]. Moreover, implementation across extensive apiary systems would likely require repeated, standardised applications, increasing logistical complexity and reducing economic feasibility relative to established control measures.

Research on *P. larvae* phages and their encoded enzymes has predominantly concentrated on their efficacy against the vegetative forms of the bacterium, with limited investigation into their impact on bacterial spores. *P. larvae* phages have demonstrated significant lytic activity against vegetative cells, but their effectiveness against the spores, known for their resilience and longevity, remains largely unexplored [59,60]. Spores are the primary etiological agent in AFB and pose a substantial challenge for eradication efforts. As phages typically target only the vegetative state of bacteria, the inability to address spores limits the overall effectiveness of phage therapy in controlling AFB. Consequently, current research highlights the need for complementary strategies that address both vegetative cells and spores to achieve comprehensive management of AFB in apiculture.

While in vitro studies on phage endolysins targeting *P. larvae* exhibit promising potential, the limited availability of these studies constrains the ability to derive definitive conclusions [59,60]. To date, a total of two lysins have been recombinantly expressed and their lytic activity assessed, with neither demonstrating activity against dormant or germinating spores [59,60]. Consequently, more comprehensive research is needed to investigate a broader array of phage lytic enzymes under both laboratory and hive conditions. Such studies would enable a detailed comparison of the full spectrum of bactericidal activities of *P. larvae* phage endolysins. Furthermore, the methods to enhance the lytic activity of these lysins should be additionally explored, specifically against spores as well as ensuring activity is present at the site of infection, being the larval gut [8,68]. Although the studies mentioned above were significant in reducing the vegetative bacterial cells, the etiological agent of *P. larvae* is its spores. In turn, therapeutic approaches must be focused on targeting these spores.

The lytic activity of endolysins is additionally highly dependent on the environment, specifically the hive environment, larval gut conditions as well as the specific characteristics and stability of the lysin [37,56]. As such a comprehensive characterisation of endolysin stability profiles, particularly in response to diverse environmental parameters such as temperature and pH present within the bee hive, is needed. These factors can significantly constrain the therapeutic potential of phage applications. To protect phages and endolysins from inactivation and maintain stability during and after application, several strategies would need to be employed, specifically against hive conditions including temperature and humidity. It is crucial that the treatments retain activity within various weather conditions as these conditions can vary within the hive throughout the year [69,70]. Although bees have mechanisms to control the hive climate, these depend on the outside weather conditions as well as the overall colony health [69]. Therefore, to protect the phages or lysins applied to control AFB, methods that are harmless to the bees that prolong the lytic activity of the treatments must be considered—such as encapsulation, the addition of stabilisers, and chemical or genetic modifications to extend activity duration and enhance resistance to environmental stressors [71]. Moreover, future research endeavours should consider assessing the optimal lytic activity of these endolysins against *P. larvae* across various growth stages to ascertain the precise lytic potential of these endolysins. While bacteriophages exhibit behaviours that should not impede the overall health of larvae, it is imperative to investigate whether the lytic ability of endolysins has any adverse effects on their microbiota and growth [35,56]. Ultimately, advancing our understanding of the interactions between phage endolysins and their environmental contexts, coupled with the development of robust stabilisation methods, is crucial for optimising phage therapy against *P. larvae*.

Phage therapy, particularly utilising bacteriophages to lyse the vegetative forms of the bacteria, has shown promise. However, these phages are more effective in prevention than treatment [50,52]. The potential of phage-encoded enzymes, such as endolysins, is particularly promising, offering a pathway to reduce the need for drastic measures such as hive destruction [59,60]. For these therapies to be viable, comprehensive research is needed to evaluate their therapeutic efficacy and safety. This research should explore various factors, specifically their effectiveness against spores, formulation compositions, preparation methods, and application techniques, both in vitro and in vivo at different stages of bee development. The successful application of phages or their enzymes in AFB treatment could bring substantial economic and environmental benefits.

### 3.3. Probiotics

The use of antibiotics in treating AFB presents certain disadvantages including concerns related to resistance and the contamination of bee products. As an alternative approach, studies have explored the use of probiotics. The term ‘probiotic’ has been used in numerous ways over the years; however, it is currently used to indicate microorganisms and their components which benefit the host by enhancing the effects of the indigenous microflora [72]. The administration of probiotics can restore the natural balance of gut microflora, thereby promoting normal nutrition and growth, and bolster immune health in animals [73]. In various animal species such as horses, mice, chickens, and fish, specific bacterial strains from the genera *Lactobacillus* and *Bifidobacterium* have demonstrated effectiveness in disease prevention and treatment [74,75]. However, direct extrapolation of these findings to honey bees is not straightforward due to the highly specialised and conserved nature of the honey bee gut microbiota, which differs markedly from that of other animals. Pioneering research has highlighted the importance of probiotics in honey bees, as outlined in Table 4, and specifically investigated novel probiotic candidates for use as an AFB therapeutic [76,77].

Among the bacterial groups most frequently found in honey bee microbiota, lactic acid bacteria (LAB) have garnered significant interest as potential probiotics [78]. Recent studies have focused on elucidating the honey bee’s response following exposure to probiotic organisms [77,79]. These investigations have considered critical aspects to be the initiation of the innate immune system and direct pathogen concentration. Probiotics play a key role in maintaining homeostasis in the event of a pathogen infection by activating the immune system [73]. However, further recent research has also demonstrated their capacity to directly counteract pathogens [77,79]. Therefore, understanding these mechanisms is essential for developing targeted and effective treatment strategies. In the pivotal study conducted by Evans and Lopez [76], honey bees were orally administered a blend of bacterial species from the genera *Lactobacillus* and *Bifidobacterium*. The researchers observed that these non-symbiotic bacteria could induce a strong immune response. Notably, the levels of the AMP abaecin were significantly elevated [76]. This observed increase in AMP abaecin levels signified an augmented immune response, facilitating a strengthened ability to combat AFB infection. Furthermore, investigations have also revealed that certain native bacterial species originating from the honey bee gut exhibit antagonistic activity against pathogens [80,81,82,83,84]. For instance, strains of *Lactobacillus* (CRL1647 and IG9) directly isolated from the bee gut and honey samples demonstrated beneficial impacts on honey bee colony health; the study particularly revealed lower indigence levels within their *Nosema* levels as well as *Varroa* occurrence, when lactobacilli were administered to the colony [85,86,87]. In 2013, Yoshiyama et al. [88] delved deeper into the potential use of LABs isolated from fermented feeds and food as probiotics to combat AFB infections. Although larvae were not directly exposed to *P. larvae* following the administration of the probiotics, the in vivo study yielded valuable insights into the interactions between isolated LABs and the larvae. Notably, the study demonstrated the LAB’s capacity to inhibit *P. larvae* growth, a finding that was linked to their strong bacterial adhesion [88]. Moreover, Daisley et al. [89] selected and combined three lactobacilli strains, Lp39, GR-1 and BR-1, and orally administered them to the larvae via a nutrient patty. The findings demonstrated a significantly lower *P. larvae* pathogen load and proteolytic activity in the larvae in the treated hives, compared to the untreated control hives [89]. In turn, the probiotic treatment was found to inhibit *P. larvae* as well as positively modulate the larvae’s innate immune response upon infection. This study was further expanded upon by the same researchers who, under normal field conditions with a naturally occurring AFB outbreak, additionally observed that administering these *Lactobacillus* strains effectively mitigated gut dysbiosis and immune dysregulation induced by the antibiotic treatment of oxytetracycline [90]. This is specifically important as it allows the authors to understand realistic disease dynamics as well as to understand the ecological impacts of the treatments.

**Table 4:** Current application of probiotics against *P. larvae* in honey bees.

Probiotic	Source	Treatment	Results	Reference
Blend of *Lactobacillus* and *Bifidobacterium* sp.	*Lactobacillus* sp., and *Bifidobacterium* sp.	Larvae fed an array of probiotic bacterial spores to assess the RNA levels of antibacterial peptides, abaecin and defensin upon *P. larvae* challenge.	Immune response was 21-fold higher in larvae exposed to probiotic spores verses the control larvae. RNA levels for abaecin were significantly higher in larvae exposed to *P. larvae*.	[76]
Various LAB strains derived from the honey stomachs of honey bees.	*Lactobacillus* sp., and *Bifidobacterium* sp.	In vitro-reared larvae were orally administered LABs individually or as a mixture originating from the honey stomach and challenged with *P. larvae* spores. Bacterial challenge occurred at various time points: pre-LAB treatment, post-LAB treatment and in conjunction with LAB.	The addition of the LAB mixture to the larval food significantly reduced the number of infected larvae in the exposure assays. The feeding time of the LAB was insignificant.	[78]
Various LAB strains isolated from fermented foods	*Enterococcus* sp., *Weissella* sp. and *Lactobacillus* sp.	Oral administration of LABs to in vitro-reared larvae. Immune activation was measured using RT-PCR to detect levels of antimicrobial peptides, abaecin, defensin and hymenoptaecin. Larvae were not challenged with *P. larvae* infection.	Nine LABs isolated from fermented food had high levels of inhibition on *P. larvae* growth in vitro. Transcriptional levels of antimicrobial peptide genes were found to have significantly increased in larvae when fed a diet containing LABs.	[88]
LX3 BioPatty—prophylactic supplementation containing Lp39, LGR-1, and LkBR-1 strains of lactobacilli	*Lactobacillus* sp.	Supplementation of probiotic lactobacilli delivered through a nutrient patty, BioPatty. Hive was exposed to *P. larvae* using a naturally occurring AFB outbreak.	Significantly lower pathogen load and proteolytic activity of larvae treated with BioPatty. An increased survival of laboratory-reared honey bee larvae in an acute infection model of *P. larvae* as weak as mitigated disease severity during the AFB outbreak.	[90]
LX3 BioPatty—prophylactic supplementation containing Lp39, LGR-1, and LkBR-1 strains of lactobacilli	*Lactobacillus* sp.	Supplementation of probiotic lactobacilli (LX3), alongside the administration of oxytetracycline (OTC) on a naturally occurring AFB outbreak. Hive was exposed to *P. larvae* using a naturally occurring AFB outbreak.	Hive supplemented with LX3 can aid in clearance rates during low-grade infection and can improve health. LX3 improved adult microbiota post-OTC exposure. LX3 supplementation suppressed *P. larvae* infection more effectively than OTC treatment alone in brood and demonstrated a capacity to lower *P. larvae* loads in gut microbiota in adult bees.	[90]
*L. apais* (HSY_B25), *L. panisapium* (PKH2_L3) & *L. melliventris* HSY_B5	*Lactobacillus* sp.	LABs isolated from honey bee gut bacteria and orally administered to larvae. Treated larvae were challenged with *P. larvae* spores at a concentration of 1000 spores/uL post-administration of LABs over 7 days.	Survival rate after 5 days of infection was between 77 and 95%. High adhesion ability of LABs to *P. larvae* also exhibited.	[79]

Recent findings expand the scope of probiotic biocontrol. Nowotnik et al. [91] evaluated six non-symbiotic bacterial species, including *Bacillus pumilus*, *B. licheniformis*, *Streptomyces narbonensis*, *Lysinibacillus fusiformis*, *Levilactobacillus brevis*, and *B. megaterium*, for their antimicrobial activity against *P. larvae* strains ATCC 9545 and CCUG 48973 via a well-diffusion assay. Notably, *S. narbonensis*, *B. licheniformis*, and *B. megaterium* demonstrated the strongest inhibition zones, while *L. brevis* and *B. pumilus* showed moderate activity [91]. These results suggest that environmental bacteria with probiotic potential may act synergistically with established LAB strains to suppress *P. larvae*, offering an expanded toolkit for AFB biocontrol. These findings highlight the potential of harnessing probiotic strains as a promising avenue for bee health management and disease prevention.

#### Limitations of Probiotics

The importance of probiotics in honey bee management has garnered increasing recognition. These beneficial microorganisms hold promise as a sustainable solution for maintaining honey bee health and mitigating infections. However, several critical aspects remain to be explored, particularly in the context of AFB. While probiotics exhibit promise, their specific efficacy against AFB demands further investigation, specifically as an understanding of the mechanisms by which probiotics interact with *P. larvae* remains unknown and is crucial for developing targeted therapeutics. Current interest centres around probiotic formulations aimed at maintaining honey bee fitness and bolstering immune functions [76,81,86]. These formulations offer a potential avenue for sustaining bee health and reducing disease incidence. However, selecting the appropriate probiotics requires rigorous evaluation, with factors such as strain specificity, dosage, and application methods needing to be considered [74,75]. Bee health is influenced by a myriad of stressors, including pesticides, pathogens, and environmental changes [18,78]. Probiotic use must be tailored to address these challenges. Unfortunately, studies in managed colonies are limited, necessitating more comprehensive research. Long-term monitoring of probiotic-treated hives is additionally essential to assess their impact on colony survival and productivity. Moreover, to optimise probiotic interventions, we must understand the intricate relationships between probiotics and native gut strains, specifically competition and potential negative interactions that need to be minimised [74,78]. Investigating these mechanisms will guide probiotic selection and deployment. Additionally, exploring the impact of probiotics on overall hive microbiota composition is vital. As the knowledge of bacterial communities continues to expand, the current state of probiotic research in honey bee health highlights the need for continued investigation into their effective use to safeguard these vital pollinators.

### 3.4. Essential Oils

Essential oils (EOs) present a promising avenue for alternative therapeutics against various bacterial pathogens. These aromatic compounds, extracted from diverse plant parts such as leaves, petals, wood, bark, resin, roots, and seeds, are commonly characterised by their sharp taste and pleasant aromatic scent [82,92,93]. While the precise mechanism of EOs’ action on plants remains to be fully explored, it is widely acknowledged that their active components exert destructive effects on target bacterial cells. Notably, EOs exhibit hydrophobic characteristics, allowing them to bind to bacterial cell membrane lipids. This interaction increases cell membrane permeability, ultimately leading to cellular damage [93].

Numerous studies have demonstrated the inhibitory effects of selected EOs on *P. larvae* [94,95,96,97]. Table 5 outlines the current application of essential oils against *P. larvae*. In their 1996 study, Alippi et al. [94] reported significant inhibition activity against *P. larvae* from the EOs lemongrass (*Cymbopogon*), thyme (*Thymus vulgaris*), and chamomile (*Matricaria chamomilla*). Similarly, Albo et al. [95] identified peppermint (*Mentha piperita*) and Andean thyme (*Acantholippia seriphioides*) as potent inhibitors of *P. larvae* growth. These works together validate the antibacterial efficacy of thyme, specifically Andean thyme, against AFB. Kuzyšinová et al. [92] highlighted oregano (*Origanum vulgare*), thyme, and clove essential oils as exhibiting strong significant inhibitory effects against *P. larvae*, contrasting with the negligible antibacterial activity of chamomile, rosemary, and fennel essential oils. Meanwhile, Kloucek et al. [98] discerned horseradish (*Armoracia rusticana*) EO as the most impactful on *P. larvae* inhibitory growth, followed by thyme and summer savory (*Satureja hortensis*). Gende et al. [99] demonstrated that cinnamon (*Cinnamomum zeylanicum*) EO exhibited a disease control potency comparable to oxytetracycline, as evidenced by both laboratory and field experiments, detailed in Table 5. However, the known allergenic and irritant properties of cinnamon essential oil highlight the need to consider safety alongside efficacy [99]. Insights from field trials revealed that, at 24 and 31 days post-treatment, AFB-challenged hives treated with cinnamon oil exhibited significantly lower incidences of infected larvae (7.89% and 52.42%) compared to the control group, indicating an efficient control mechanism [99]. These findings serve as compelling evidence of cinnamon oil’s potential in effectively managing AFB, while posing minimal toxicological risks to bees [99]. In additional investigations, Pellegrini et al. [97] and Albo et al. [95] scrutinised various EOs for their antibacterial activity against *P. larvae*, revealing widespread antibacterial action, except for chilca (*Baccharis latifolia*) EO, which lacked such efficacy, thereby causing no damage to the cell wall and cytoplasmic membrane of *P. larvae*.

Regarding their impact on honey bees, Ablo et al. [95] established the median lethal dose (LD50) values for thyme, lemongrass (*Cymbopogon citatus*), oregano, and basil extracts (*Ocimun basilicum*), revealing minimal toxicity levels. However, their investigation found that even though these extracts showed low toxicity, field trials demonstrated that neither individual essential oils nor their blends effectively eliminated AFB’s clinical symptoms, regardless of the dose formulation or method of administration tested.

Although not an essential oil but rather a plant-derived compound, one investigation [100] into crude ethanolic extracts (CEEs) from the leaves of *Trapa bispinosa* Roxb (TB) and *Euryale ferox* Salisb (EF), along with fractions obtained via liquid–liquid extraction using petroleum ether, ethyl acetate (EA), and *n*-butanol, demonstrated significant in vitro growth-inhibitory activity against a local strain of *P. larvae* LC–MS analysis-identified gallic acid (GA) as the most abundant compound in the EA fraction of TB [100]. GA exhibited potent antibacterial activity against *P. larvae*, with Minimum Inhibitory Concentration (MIC) and Minimum Bacterial Concentration (MBC) values of 125 and 250 μg/mL, respectively, and acted through a cell membrane-disturbing mechanism [100]. Importantly, GA also inhibited spore germination, a critical step in AFB pathogenesis. This dual action, blocking vegetative growth and spore germination, positions GA as a promising candidate for AFB management.

#### Limitations of Essential Oils

Treating AFB with EOs presents a multifaceted array of challenges and considerations. Notably, many of the studies mentioned above have not conducted challenges with *P. larvae* infections post-EO treatment. This gap in research leaves unanswered questions regarding the effectiveness of essential oils in mitigating actual *P. larvae* infections in honey bee colonies. While the inhibitory effects of certain essential oils on *P. larvae* growth, such as cinnamon, thyme and oregano, have been demonstrated in vitro [95,96,99], their efficacy in real-world scenarios where bees are exposed to natural environmental conditions and potential recontamination remains uncertain. Consequently, further investigations incorporating challenge experiments under field conditions are warranted to ascertain the practical utility of essential oils as a viable therapeutic option for managing AFB in honey bee colonies. Additionally, elucidating the intricate dynamics of how essential oils interact with the native gut microbiota of bees poses a significant scientific hurdle [83]. The complex composition of EOs and their potential impact on the delicate balance of the bee gut microbiome require thorough investigation to ensure both therapeutic efficacy and minimal disruption to the bees’ health [83,95]. While EOs are often touted for their natural properties, concerns persist regarding their influence on honey characteristics, including aroma, flavour, and even potential antimicrobial properties within the hive [82,92]. Moreover, the application of essential oil-based solutions, particularly in gaseous form, may introduce novel environmental stressors to bee colonies, potentially affecting their flight behaviour, foraging patterns, and overall social dynamics [69]. Furthermore, incorporating essential oils into bee feed raises questions about palatability alterations, potential impacts on colony acceptance, and long-term effects on bee health [69,95]. Additionally, while gallic acid shows promising antibacterial and spore-inhibitory properties, several limitations must be considered. Its efficacy can vary significantly among *P. larvae* strains, with reported MIC values ranging from 62.5 μg/mL to 2500 μg/mL, indicating inconsistent performance across genotypes [100,101,102]. Additionally, the precise mechanism of action remains unclear, which complicates optimisation and risk assessment. Gallic acid’s stability under hive conditions, potential interactions with bee microbiota, and effects on honey quality have not been fully evaluated. Addressing these multifaceted challenges necessitates a combined approach, encompassing rigorous scientific inquiry, stringent quality control measures, and comprehensive assessments of both short-term efficacy and long-term implications for honey bee colonies and apiary ecosystems.

## 4. Other Controls

Considerable effort has been devoted to developing sustainable treatment strategies for AFB, encompassing approaches at varying stages of research and application. New antibiotics face ongoing challenges in hive use, while antagonistic bacteria targeting *P. larvae* in larvae are currently in early experimental stages [79,89]. Essential oils (EOs) have shown promise in laboratory studies, though their field efficacy and potential effects on bees require further evaluation [96,101]. Other lesser-investigated treatments that have been considered include small molecules, fatty acids, and breeding for hygienic traits, which offer additional avenues for future control strategies.

### 4.1. Small Molecules

The eradication of AFB remains challenging because *P. larvae* spores exhibit exceptional resistance to high temperatures, desiccation, UV irradiation, and harsh chemicals. These spores can persist in various components of the hive, including honey, pollen, wax, adult bees, and hive surfaces, allowing long-term contamination [40,68]. Although antibiotics have historically been used to treat active infections and prevent AFB, they do not affect dormant spores, which can survive well beyond treatment [5,40,68]. Since spore germination is the initial step in infection, targeting this process represents a promising strategy for disease prevention. Similar approaches have been successfully applied to inhibit spore germination in pathogens such as *Bacillus anthracis* and *Clostridium difficile* [103,104]. Building on this, small molecules have gained attention as alternative therapeutics for *P. larvae*, targeting various stages of infection. Previous research identified uric acid and L-tyrosine as co-germinants essential for *P. larvae* spore germination, and indole derivatives such as 5-chloroindole, 5-bromoindole, and 5-nitroindole demonstrated strong inhibitory activity with IC_50_ values significantly lower than indole itself, while exhibiting minimal antibiotic effects [103,104]. These compounds not only suppressed germination in vitro but also protected honey bee larvae without toxicity, making them attractive candidates for non-antibiotic AFB control. However, their antibacterial effect on vegetative cells is modest, representing a major limitation; their stability under hive conditions requires further validation. Complementing germination inhibitors, anti-virulence strategies have targeted Plx2A, an ADP-ribosylating exotoxin implicated in larval cell damage [103,104]. A synthetic pyrazolo-pyrimidine compound (M3) inhibited Plx2A enzymatic activity while two plant-derived flavonoids (acacetin and baicalein) were even more potent [104]. These molecules prevented toxin-induced cytoskeletal disruption in insect cell cultures, yet failed to reduce larval mortality in infection models, suggesting that neutralising a single virulence factor is insufficient against AFB’s multifactorial pathogenesis. Flavonoids also face challenges related to solubility, hive stability, and potential microbiome impacts [103]. Meanwhile, indole analogues act at the earliest stage of infection by preventing spore germination, effectively blocking disease initiation. In contrast, the synthetic compound M3 targets downstream virulence mechanisms, specifically inhibiting the Plx2A exotoxin. Each strategy offers distinct advantages; indoles excel in halting infection before it begins and flavonoids provide toxin-specific inhibition, but no single approach of these small molecules currently addresses the multifactorial nature of AFB pathogenesis.

### 4.2. Fatty Acids

Fatty acids (FAs) represent a further promising alternative approach to positively impacting bee health. A key attribute of FAs lies in their capacity to incorporate themselves into membrane lipids, thereby modulating the permeability of cell membranes and disrupting cellular homeostasis, ultimately leading to cell demise [105]. Moreover, FAs assume a pivotal role in bee nutrition, sourced primarily from pollen, which serves as a vital component of the larval diet along with royal jelly [106,107]. Early research by Rinderer et al. [106] proposed a correlation between larval intestinal pollen content and their resistance to AFB. Larvae receiving pollen supplementation prior to AFB exposure exhibited a 20% reduction in mortality compared to control counterparts [106]. Hornitzky [108] further explored the potential of 28 individual FAs as AFB control agents, identifying significant antibacterial activity in 15 of them, including undecanoic, lauric and linoleic acid. Despite these promising findings, subsequent investigations have not subjected these fatty acids to bio-exposure assays or evaluated their efficacy or specificity within bee colonies. In turn, progress in leveraging FAs as therapeutic agents has stalled since 2003, with this avenue of research remaining largely unexplored.

### 4.3. Breeding for Hygienic Traits

The hygienic behaviour of honey bees has been subjected to thorough scrutiny for its potential role in bolstering resistance against pathogens. This behaviour is characterised by the meticulous uncapping and removal of compromised brood cells. Rothenbuhler and Seltzer et al. [26,109] initially garnered scientific attention for a potential mechanism for combatting AFB. Early investigations by Rothenbuhler [26] delved into the intricacies of this behaviour, particularly its efficacy in AFB management. Central to the effectiveness of hygienic behaviour is its ability to expediently remove infected pupae before the pathogenic bacteria reach a transmissible stage [110]. While AFB-resistant traits were successfully cultivated at a heightened frequency within a government-maintained (Ohio, USA) breeding population between 1935 and 1949, the integration of these traits into commercial breeding programmes remains elusive [26]. Despite consensus regarding the efficacy of hygienic behaviour in reducing brood diseases, there exists uncertainty regarding its scalability within commercial bee populations without prior optimisation in controlled environments [26]. To date, the low occurrence of the trait has resulted from selective breeding in small, closed, experimental populations [26,111]. Moreover, the low heritability of hygienic behaviour, as reported by Pernal et al. [111], with estimates ranging from 0.17 to 0.25, further constrains its potential for improvement through selective breeding. These findings merit the exploration of alternative AFB-resistant traits with higher heritability, such as polygenic resistance or traits identifiable through genomic selection, which may provide a more robust and effective approach to infection management.

## 5. Application of Current Avenues for AFB Mitigation

The management of AFB in honey bees necessitates an integrated approach combining both traditional and emerging strategies. Current methods, such as the use of probiotics and essential oils, have shown potential for improving overall colony health and resilience. However, their efficacy against *P. larvae* is limited by their non-specific modes of action. Additionally, these treatments are heavily reliant on supplemental feeding for delivery, typically via sugar syrups or protein patties [38,91,99]. Supplemental feeding provides a practical vehicle for therapeutic administration but is constrained by seasonal factors, as bees often reject supplementary diets during periods of natural forage abundance [112]. This limits the continuous application of these treatments and, consequently, their long-term effectiveness. Similarly, bacteriophage therapy, a promising pathogen-specific biocontrol method, also depends on supplemental feeding or hive-based application systems to deliver phages to the target pathogen. While bacteriophages offer unparalleled specificity in lysing *P. larvae*, their success hinges on consistent and effective delivery mechanisms to ensure adequate uptake and persistence within the hive environment [7,56,113]. The reliance on supplemental feeding as a primary delivery method underscores the need for more innovative and autonomous systems that mitigate the challenges associated with seasonal feeding practices. Among emerging strategies, TGIP offers a significant advantage by circumventing the limitations of supplemental feeding. TGIP-based vaccines utilise the natural ability of queen bees to pass pathogen-specific immunity to their offspring, providing colony-wide and long-term resistance without the need for repeated external administration [27,31]. This approach aligns with the bees’ life cycle and enhances both colony resilience and the specificity of the immune response against a variety of pathogens.

The future of AFB management likely lies in a multifaceted approach, integrating TGIP-based vaccines with other strategies, including bacteriophages, probiotics, essential oils, and supplemental feeding during critical periods. This synergistic framework would leverage the strengths of each method while addressing the logistical challenges of delivery systems. Looking ahead, the future of AFB management is filled with promise. Continued research into improving delivery methods, especially for phages and TGIP-based vaccines, and understanding the synergistic effects of combined treatments, holds great potential. By embracing these innovative strategies, it is possible to develop sustainable, effective solutions that protect honey bee populations and ensure their vital role in global agriculture and ecosystems.

## 6. Conclusions

While honey bees stand as one of the most economically valuable pollinators, their population faces a myriad of threats from various pathogens. Among these, AFB poses a particularly grave danger, being highly virulent, contagious, and most often fatal to honey bee larvae. Despite the urgency, there remains a critical gap in the availability of safe and effective prophylactic solutions for disease prevention and treatment. Presently, only one option stands as a commercial product, namely the Dalan Animal Health (Athens, GA, USA) vaccine. However, the current body of literature reflects a consensus that alternative methods of treatment are lacking in efficacy and require immediate attention to meet the pressing needs of agricultural and livestock management. Urgent action and innovation are imperative to safeguard honey bee populations and ensure the sustainability of pollination services vital for global food security.

## Figures and Tables

**Table 2:** Current application of *P. larvae* phages in honey bees.

Phage Name	Phage Source	Treatment	Results	Reference
HB10c2 phage	Isolated from glue-like liquid within a beehive exhibiting clinical symptoms caused by *P. larvae* ERIC I.	In vivo, phage therapy was conducted via feeding using a diet containing *P. larvae* spores (500 cfu/larvae) and bacteriophages (50,000 cfu/larvae).	Phage had no harmful impact on the survival of bee larvae. Mortality rate was significant with treatment. Application of phage did not exhibit therapeutic effects against AFB.	[50]
F, WA and XIII phage	Phages isolated from various samples. F Phage -*P. larvae* strain 2231, W Phage—soil under hive and XIII Phage—infected hive scale.	Larvae were infected with *P. larvae* strain NRRL B-3650 spores, then treated with either single phage or phage cocktail and assessed for 8 days.	Phage treatment had no deleterious effect on larvae survival. Prophylactic treatment with phages saw a higher rate of survival when administered after infection.	[46]
Phage cocktail: 1, 5 and 9	Phages grown and isolated from bacterial strain *P. larvae* ATCC 9545.	Healthy hives were given a phage cocktail through feeders or via a spray. *P. larvae* challenge was performed 2 weeks post-treatment. Hive inspections were performed to assess AFB infection level.	No statistical difference observed in bee deaths with phage application. Results noted that dosing levels even at levels of overdosing bees with phages did not negatively impact uninfected hives. Protective and therapeutic effects were observed.	[51]
Phage cocktail: Xenia, Halcyone, Willow, Fern, Vadim, Harrison, and Hayley	Various sources of isolation: Xenia—scale from infected hive, Halcyone—propolis, Willow/Harrison/Hayley—soil under hives, Fern—wild strain 2231 and Vadim—lip balm	Phage cocktail containing titre of 1.8 × 10^6^ cfu/larvae administered orally either before or after infection of spores. Treatment dose increased over a period of 7 days.	Results indicated regardless of the time the phages were administered (pre- or post-challenge) the survival of larvae significantly increased by approximately 59% when compared to the untreated control.	[52]

**Table 3:** Current application of *P. larvae* phage endolysins in honey bees.

Endolysin Name	Source	Treatment	Results	Reference
PlyPI23	Isolated from genome of phiIBB_Pl23.	Diet with 2.0 µM of endolysin was fed to larvae over 5 days. No spore challenge was performed.	No adverse effects on larvae were observed.	[59]
PlyPalA Lysin	Isolated from genome of Xenia phage. Xenia phage isolated from an environment sample.	Larvae were infected with *P. larvae* B-3650 spores during feeding then orally administered the endolysin incorporated into the feed at a concentration of 16 µg/mL.	Larvae survival rate after spore challenge increased from 23% to 75% when treated with the endolysin.	[60]

**Table 5:** Current application of essential oils (EOs) against *P. larvae* in honey bees.

Essential Oil	Treatment	Results	Reference
Savory, thyme, lemongrass and oregano	Oral administration of EOs at lethal dose 50 (LD50) of pure essential oils as well as a blend of essences.	Field trials indicated neither individual EOs, nor the blends, were effective in eliminating AFB clinical symptoms at any dose formulation or method of administration tested.	[95]
Cinnamon	Three weekly doses via oral administration of EOs in an apiary field trial. Hive was then challenged with AFB. Evaluation of AFB disease incidence was made by counting the number of infected brood cells weekly.	After 24 and 31 days from the beginning of treatments, the EO-treated hives showed a lesser incidence of infected larvae (7.89% and 52.42%) than the control group. Highlighted a clear efficient control with minor toxicological risks to bees.	[100]
Andiroba and Copaiba	Bio-exposure assay was not performed. Bee morality rates and inhibitory rates of *P. larvae* growth as a result of EO treatment were performed separately. Bees were sprayed with oil and morality rates were examined for 10 days.	No bee morality in bees treated with Copaiba oil after 10 days observation. After 24 h treatment with Andiroba or Copaiba oil, no viable cells of *P. larvae* were observed. Copaiba oil good candidate for treatment or preventative measure for AFB.	[96]

## Data Availability

No new data were created or analysed in this study. Data sharing is not applicable to this article.

## Abbreviations

The following abbreviations are used in this manuscript:

AFB	American Foulbrood
EO	Essential Oils
FAs	Fatty Acids
LAB	Lactic Acid Bacteria
Phages	Bacteriophages
TGIP	Transgenerational Immune Priming
Vg	Vitellogenin
